# Influenza A Virus Utilizes the Nasolacrimal System to Establish Respiratory Infection after Ocular Exposure in the Swine Model

**DOI:** 10.1155/2024/8192499

**Published:** 2024-06-27

**Authors:** Shubin Li, Xuebin Peng, MinJie Wang, Wenqian Wang, Yuye Liu, Qian Yang

**Affiliations:** MOE Joint International Research Laboratory of Animal Health and Food Safety College of Veterinary Medicine Nanjing Agricultural University, Nanjing, Jiangsu, China

## Abstract

Influenza A virus (IAV) can rapidly disseminate among animals through various transmission routes, with emerging evidence suggesting the ocular surface as an important entrance. However, it remains unclear how the virus invades the respiratory tract after ocular exposure. Here, we demonstrated that H1N1 (A/swine/Guangdong/1/2011) utilizes the nasolacrimal system to rapidly spread from the ocular surface to the respiratory tract in the porcine model. *In vivo* and *ex vivo*, IAV could efficiently attach and replicate in conjunctiva epithelium, which has abundance of *α*-2,6-linked and *α*-2,3-linked sialic acid. After ocular inoculation, infectious virions swiftly migrate to the nasolacrimal duct of piglets and, via continual drainage, disseminate to the respiratory tract. Moreover, the detection of continual virus shedding as well as the successful isolation of virus from conjunctiva and respiratory tract tissue indicated the establishment of productive infection after the transocular route. This study presents evidence suggesting that IAVs could utilize the nasolacrimal system to swiftly spread to the respiratory tract following ocular exposure, which contributes to understanding the modes of transocular transmission of IAVs.

## 1. Introduction

The pandemic caused by the influenza A virus (IAV) poses a serious public health threat worldwide [1]. Typically, IAVs spread rapidly among animal populations primarily through the respiratory tract [2]. Recent studies have increasingly highlighted ocular infection as an alternative route of viral entry [3, 4, 5, 6]. Previous reports showed that the H7 subtype IAV possesses an apparent ocular tropism in humans, leading to conjunctivitis and blindness in some cases [7, 8]. Meanwhile, ocular complications have been documented in patients infected with human H1N1 and avian H5N1 [7, 9, 10, 11]. Many studies have shown that multiple subtypes of IAVs can replicate in human ocular *ex vivo* cultures or primary cells [12, 13, 14]. Chan and her colleagues demonstrated that the surface proteins (hemagglutinin and neuraminidase) participate in the conjunctival tropism difference of H1N1pdm and seasonal H1N1 [12]. Moreover, the tears could significantly inhibit the hemagglutination of IAVs with lesser effect on ocular isolates than nonocular isolates [15]. There is still a need for further studies to elucidate precise molecular mechanisms underlying ocular tropism [13, 16]. Transocular infection was observed during the 2009 H1N1 pandemic and the recent COVID-19 pandemic highlighting the risk of nonrespiratory transmission of the respiratory viruses [17, 18]. Therefore, understanding the modes of transocular transmission is crucial for controlling viral infection in the future pandemic.

The ocular surface comprises a continuous epithelium consisting of conjunctiva, cornea, and nictitating membrane (vestigial in humans), representing a mucosal barrier that is directly exposed to the external environment [6]. Epithelial cells of conjunctival and corneal with viral receptors can act as direct entry sites for infectious virions contained in aerosols [3]. It is worth noting that humans and animals have exhibited typical respiratory syndrome (cough, sore throat, and nasal congestion) following ocular exposure to IAVs [16, 19]. Belser and colleagues found that both avian and mammalian IAV could establish respiratory infection in ferrets following ocular inoculation [16]. Applied special equipment, ocular-only exposure to virus-containing aerosols caused fatal respiratory infection in ferrets, even for the strains that do not determine an ocular tropism [20]. Moreover, IAV caused a productive and transmissible infection in ferrets following transocular inoculation, with no difference in peak viral titers and release of virus-containing aerosols compared with intranasal infection [21]. A comprehensive assessment of how ocular exposure modulates the transmissibility of IAVs is important [19]. Withal, the mechanisms contributing to respiratory infection after ocular exposure have not been elucidated. The nasolacrimal duct, which connects the ocular surface to the nasal passage, plays a role in the rapid drainage of tear film [17, 22]. Given its anatomic characteristics, the nasolacrimal duct probably harbors and transports the virus to other tissues following ocular exposure, while the relative studies are still much less.

Due to the expression of both *α*-2,3-linked (avian-type receptor) and *α*-2,6-linked (human-type receptor) sialic acid (SA), swine are vital intermediate hosts for the emergence of novel influenza viruses [23]. Swine possesses the same sensitive subtype IAVs (H1N1, H1N2, and H3N2) and resembles infection mechanisms with humans, making them a promising animal model for investigating IAV infection [24, 25]. Moreover, the ocular tissue of pigs exhibits greater similarities with human beings, which contributes to the porcine, widely used in eye studies than other animal models [26]. Understanding the details of the distinct transmission routes of IAVs, particularly the nonrespiratory route, is crucial for clinical prevention and diagnosis [19]. Therefore, using the pig model to study how IAVs transmit from the ocular surface to the respiratory tract has a great referential value for humans [25].

In the present study, we investigated the distribution of both *α*-2,3-linked and *α*-2,6-linked SA in the ocular surface and respiratory tract. Piglets were inoculated with H1N1 (A/swine/Guangdong/1/2011) via ocular exposure. Viral antigens were detected not only in ocular tissue and nasolacrimal duct but also in the respiratory tract and blood cells. The *ex vivo* cultures of conjunctiva supported the productive infection by both mammal and avian influenza viruses. Furthermore, persistent virus shedding was observed in nasal swabs, and the virus was isolated from organs of the inoculated piglets at 7 days postinoculation (dpi). These studies provided evidence that H1N1 could establish a productive infection in the porcine conjunctiva and utilized the nasolacrimal duct for rapid transfer to the respiratory tract.

## 2. Materials and Methods

### 2.1. Cells and Viruses

Madin Darby canine Kidney (MDCK, purchased from ATCC) cells were cultured in Dulbecco's modified Eagle's medium (DMEM; Biochannel, BC-M-005, China) supplemented with 10% fetal bovine serum (FBS; Biochannel, BC-SE-FBS08, China) in the incubator under the condition of 5% CO_2_ at 37°C. H1N1 (A/swine/Guangdong/1/2011, TCID_50_ = 5 × 10^4^/mL, GenBank: MT410579) is a classical swine influenza virus that was kindly provided by Zhixin Feng (Jiangsu Academy of Agricultural Sciences). H9N2 (A/duck/Nanjing/01/1999, TCID_50_ = 5 × 10^5^ /mL, GenBank: DQ681221) was stored in our lab. The influenza viruses were injected into the allantoic cavity of 10-day-old SPF chicken embryos, incubated at 37°C and 75% humidity for 72 hr. Then, the allantoic liquid collected from eggs was clarified by centrifugation (3,000 rpm for 10 min) and frozen in aliquots at −80°C. After harvesting, the allantoic fluid was subjected to hemagglutination (HA) test. The RNA of virus stocks were extracted using a TIANamp Virus RNA Kit (TIANGEN, DP315, China) and used for reverse transcription (Vazyme, R302, China) for reverse transcription polymerase chain reaction (RT-PCR) targeting HA gene (600 bp), and finally confirmed by Sanger sequencing. The primer sequences were as follows: 5′-TCCACCTACCAGTGCTGACCAAC-3′ (forward) and 5′- TGCTCTTTCGGTCGGCTGCATA-3′ (reverse). The virus stock was titrated by tissue culture infectious dose 50 (TCID_50_) assay in MDCK cells according to the Reed and Muench formula.

### 2.2. Animal Experiments

The animal studies were approved by the Institutional Animal Care and Use Committee of Nanjing Agricultural University and followed National Institutes of Health guidelines for the performance of animal experiments. Sixteen 4-week-old, influenza seronegative piglets (Duroc × Landrace × Yorkshire) were obtained from a swine farm in Nanjing city. To investigate the anatomical structure of the nasolacrimal, one of the piglets was sacrificed, and then the trypan blue dye was injected into the upper nasolacrimal point of the piglet. Subsequently, the nasal cavity tissue was collected as the previous description [27]. The other animals were randomly separated into three groups (five pigs per group). Piglets in the first group (*n* = 5) and the second group were transocular inoculated with 50 *μ*L H1N1 stock containing 5 × 10^4^ TCID_50_/mL. Piglets in the third group (*n* = 5) were inoculated with 50 *μ*L PBS to serve as mock inoculation.

To value the virus transmission via the nasolacrimal duct, nasal swabs were collected from the first and third groups at 0, 5, 30, 60, 120, and 360 min after ocular inoculation. The swab should be inserted in the nasal vestibule which is the exit of the nasolacrimal duct and thrown into a centrifuge tube containing 500 *μ*L PBS [28]. The collected swabs were saved at −80°C before analysis. Moreover, anticoagulated whole blood was collected at 0, 0.5, 2, 4, and 6 hours postinoculation (hpi), and the hemocyte was collected by centrifugation for further RT-PCR targeting HA gene and western blot analysis. Then, animals from the first group were euthanized at 6 hpi, and tissue samples including eye, nasal cavity, pharyngeal, trachea, and lung were collected and immediately frozen on dry ice or fixed in 4% paraformaldehyde. Animals from the second and third groups were monitored daily for clinical signs and rectal temperature. Nasal swabs were collected daily for the quantitative real-time PCR (qRT-PCR) analyses of virus shedding as above. All the animals were euthanized at 7 dpi, and tissue samples were collected as above.

### 2.3. Viral RNA Detection and Virus Isolated

Viral RNA in nasal swabs was extracted using a TIANamp Virus RNA Kit (TIANGEN, DP315, China) according to the manufacturer's introduction and reverse transcribed into cDNA using the HiScript III RT SuperMix for qPCR (+gDNA wiper; Vazyme, R323, China). qRT-PCR was performed with a ChamQ SYBR qPCR Master Mix (Vazyme, Q711, China) using an influenza A virus M gene primer set to determine virus load. The primer sequences were as follows: 5′-ATCCTGTCACCTCTGACTAAGGG-3′ (forward) and 5′-TCTACGCTGCAGTCCTCGC-3′ (reverse). Threshold cycle (Ct) values lower than 30 were considered positive. Samples in which fluorescence was undetectable were considered negative.

Tissue specimens from ocular inoculated piglets were homogenized in 1 mL cold PBS using tissue grinders and clarified by centrifugation before filter sterilized with a 0.45 *μ*m filter. Fifty microlitres tissue homogenate of the conjunctiva, nasal mucosa, trachea, and lung were injected into eggs to isolate the virus. All the allantoic liquids were collected from eggs after 72 hr incubation and tested by hemagglutinin.

### 2.4. Histological Analysis

After fixation, samples were embedded in paraffin. For the nasal cavity, the tissues were decalcified using a 5% formic acid solution (90 mL PBS, 5 mL 4% paraformaldehyde solution, and 5 mL formic acid) for 1–2 weeks at room temperature. Five cross-sectional blocks of the nose were generated before embedding as previously described [27]. For the ocular sample, the eyes including the conjunctiva and cornea were integrally separated from the orbit and cut in half through a midsagittal plane before fixation. After embedding in paraffin, 5*μ*m thick sections were cut on a microtome (Leica, RM2015, Germany) and mounted on slides before hematoxylin/eosin (HE) staining. All the images were observed using a BX51 Digital Camera System (Olympus Inc., Japan).

### 2.5. Immunohistochemical (IHC) and Lectin Staining

The IHC staining was performed as previously described. In brief, paraffin sections were performed deparaffinization and rehydration. Then, 3% hydrogen peroxide and 5% bovine serum albumin were successively used to block nonspecific staining. Then, sections were incubated with mouse anti-HA monoclonal antibody (homemade) overnight at 4°C. Subsequently, sections were treated with streptavidin–biotin complex (SABC; BOSTER, SA1026, China) according to the manufacturer's protocol. Positive cells were visualized by treatment with diaminobenzidine (DAB; BOSTER, AR1027, China). The sections from the mock infection group were used as a negative control.

Lectin labeling was performed as previously described [14]. In brief, biotinylated *Sambucus nigra* agglutinin (SNA; Vector Laboratories, B1305-2, America) and *Maackia amurensis* lectin II (MAL II; Vector Laboratories, B1265-1, America) were used to label the *α*-2,6-linked and *α*-2,3-linked SA, respectively. Tissue sections were treated by the same procedure as IHC till the blocking steps. Then, sections were labeled by SNA (5 *μ*g/mL) or MAL II (5 *μ*g/mL) overnight at 4°C according to the manufacturer's instruction. Finally, sections were treated with SABC and DAB as described above. Negative control sections were pretreated with 5 mU of neuraminidase (Sigma, N8271, America) at 37°C for 3 hr to remove the Sia as previous description [29, 30].

All sections were counterstained with hematoxylin and images were observed using a BX51 Digital Camera System (Olympus Inc., Japan) and imaged on a microscope scanner (Grundium Ocus®40, Finland). The labeling slides were measured with integrated optical density (IOD) using Image-Pro Plus software. Staining sections from at least three different tissue depths were used to assess the IOD.

### 2.6. *Ex Vivo* Conjunctiva Cultures and Influenza Virus Infection

Four-week-old, influenza seronegative piglets were sacrificed to obtain the conjunctiva tissue. The conjunctiva was divided into four fragments and cultured in Roswell Park Memorial Institute 1640 (RPMI-1640; BC-M-017, Biochannel, China) medium containing 2% FBS, gentamicin (50 *μ*g/mL, Sanggon Biotech, China), cefixime (100 *μ*g/mL, C6570, Solarbio, China), and Penicillin/Streptomycin (100x, P1400, Solarbio, China). For infection, supernatants of *ex vivo* culture were removed and rinsed by fresh RPMI-1640 medium three times. Then, tissue cultures were incubated with the 600 *μ*L RPMI-1640 containing 5 × 10^3^ TCID_50_ of H1N1 or H9N2 at 37°C for 2 hr. Discarding the supernatants, the tissue cultures were washed three times and supplied with RPMI-1640 medium containing 2% FBS and antibiotic to continue the culture. The *ex vivo* culture (*n* = 3) was collected at 6, 12, and 24 hr after infection which were divided into two pieces for western blot and IHC.

### 2.7. Western Blot Analysis

Tissue samples were lysed using RIPA lysis buffer (Biosharp, BL504A, China) adding PMSF (BioFroxx, 9087-70-1, China). Bicinchoninic acid (Vazyme, E211-01, China) assays were used to determine the protein content. Proteins were separated by SDS-PAGE and transferred onto polyvinylidene difluoride membranes. After blocking with 5% skim milk, the membranes were explored with mouse anti-H1N1-HA monoclonal antibody (homemade) or rabbit anti-H9N2-HA antibody (Sino Biological, 11229-V08H, China). *β*-actin-HRP conjugated (Abways, AB2001, China) was used as the internal control to evaluate the protein amounts by using ImageJ.

### 2.8. Plaque Assay

MDCK cells were seeded at 5 × 10^5^ /well in 12-well plates and grew into a monolayer. The monolayers were incubated with supernatant collected from *ex vivo* culture from different time points. After 2 hr of incubation, the supernatants were discarded, and monolayers were overlaid with 0.7% low melting point agarose in DMEM containing 2% FBS, 10 *μ*g/mL TPCK trypsin (Gibco, 15050-065, America) and penicillin/streptomycin (100x). After 48 hr incubation, monolayers were stained with crystal violet to observe the plaque after removing the overlaid gel.

### 2.9. Statistical Analysis

The IOD of stained sections (*n* = 3) and the gray value of the western blot were analyzed using Image-Pro Plus 6.0 (Media Cybernetics, Inc.) [31]. All the results were determined as means and standard deviations (SD), and the statistical analysis was performed using Graphpad Prism 8. Statistical significance was determined by one-way analysis of variance (ANOVA) followed by *t*-tests. For all analyses, a *P* value of <0.05 was considered statistically significant.

## 3. Results

### 3.1. Anatomical and Histological Characteristics of Porcine Nasolacrimal Duct

**Figure 1:**
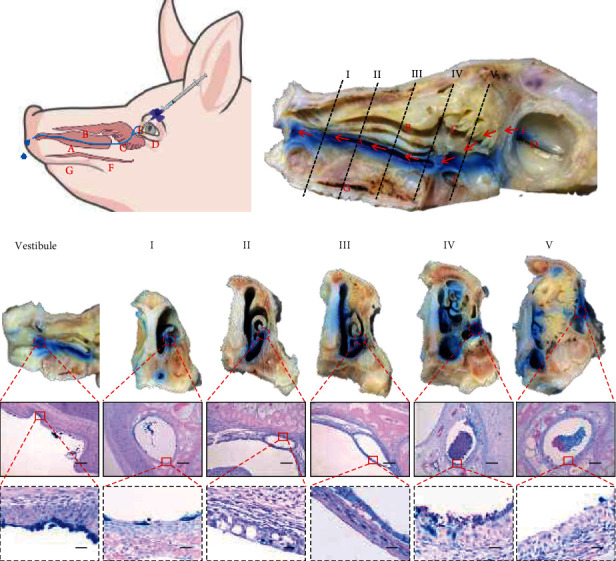
Anatomical structure and morphology of porcine nasolacrimal duct. (a) The blue line in the diagram represents the nasolacrimal duct from eye to nose. (b) The lacrimal point of the piglet was injected with trypan blue. Then, the mandible and eyeball were removed before fixation and decalcification. To better observe the duct, the ventromedial spiral of the inferior nasal concha was removed carefully. The red arrows represent the position of the nasolacrimal duct, which was colored by trypan blue. (A–G) were the positions of the porcine nasal passage: (A) Inferior nasal concha; (B) Superior nasal concha; (C) Region olfactory; (D) Eyeball; (E) Lacrimal point; (F) Nasal meatus; and (G) Hard palate. (c) The nasolacrimal duct in different cross-sections was stained by HE. The circle boxes represent HE staining of the nasolacrimal duct in different cross-sections. Bar, 200 *μ*m. The square boxes enlarge the image of the epithelium of the nasolacrimal duct. Bar, 20 *μ*m.

**Figure 2:**
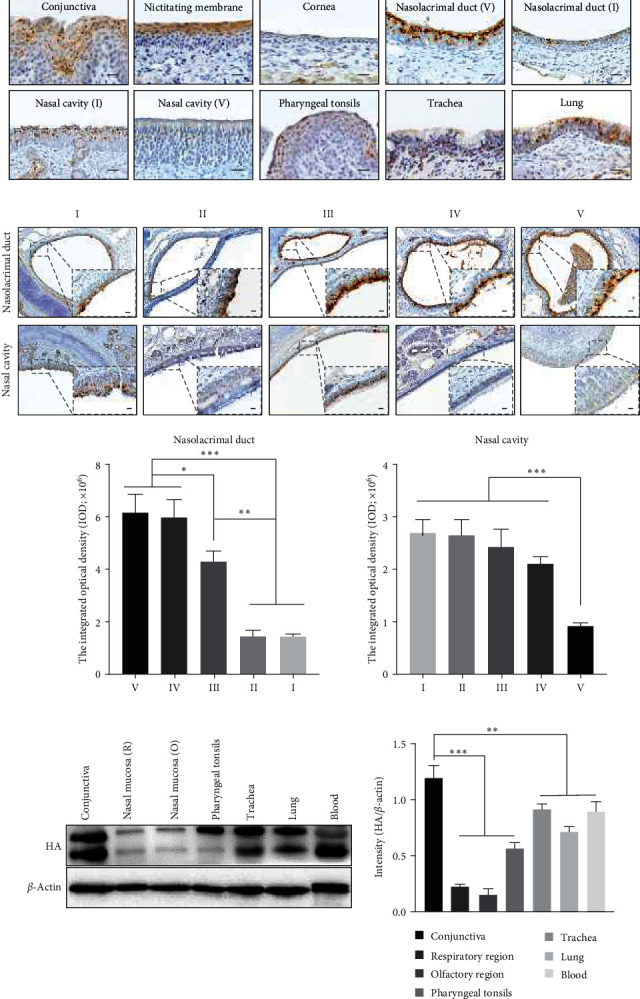
The nasolacrimal system serves as a route for virus transmission. Piglets were inoculated by H1N1 via the ocular route and sacrificed at 6 hpi. (a) IHC targeting the viral HA protein was performed in ocular or respiratory tract tissues collected from ocular-inoculated pigs. (b) IHC was performed to detect viral antigens in distinct regions (I–V) of the nasolacrimal duct and nasal passage. Bar, 10 *μ*m. The average integrated optical density (IOD) of viral HA-protein was obtained by analyzing three panoramic scans of different depth slides using ImageJ. (c) Western blot analysis of tissue samples collected from ocular-inoculated pigs, and the intensity (HA/*β*-actin) was measured using ImageJ. Two bands represent the HA0 (upper) and HA1 (lower), respectively, which might be caused by host proteinase. All data shown are the mean results ± SD from three independent experiments. Statistical significance was using one-way ANOVA. NS, no significance.  ^*∗*^*P*  < 0.05,  ^*∗∗*^*P*  < 0.01,  ^*∗∗∗*^*P*  < 0.001.

To investigate the anatomical characteristics of the swine nasolacrimal duct, trypan blue was injected from the superior lacrimal punctum using an indwelling needle (Figure 1(a)). To observe the nasolacrimal duct, the nasal septum and the inferior turbinate were removed carefully after decalcification. As the colored tissue shows the nasolacrimal duct links the ocular surface to the nasal passages and ends in the vestibule (Figure 2(b)). Furthermore, HE staining was performed in different cross sections (regions I–V) to reveal the morphology of the nasolacrimal duct. Unlike the nasal passage, only a few goblet and ciliated cells were observed in the nasolacrimal ducts (Figure 1(c)). In addition, it could be observed that the wall of the nasolacrimal duct contained abundant vascular plexuses (Figure 1(c)). The upper parts (regions IV and V) of the nasolacrimal duct are embedded in osseous tissue, while the middle parts (regions II and III) are exposed on the surface of the ventral inferior turbinate cling to the nasal mucosa. In the lower parts (region I), the exit of the duct could be observed in the vestibule.

### 3.2. IAV Invaded the Respiratory Tract after Ocular Inoculation via the Nasolacrimal Duct

To investigate the role of the nasolacrimal duct during transocular infection, piglets were inoculated with H1N1 via the ocular route and sacrificed 6 hpi. The presence of viral RNA in nasal swabs was detected at 5 min after inoculation using qRT-PCR and decreased over time (5–360 min) (Table 1). Furthermore, immunohistochemical (IHC) showed that viral antigens could be observed both in the ocular tissue and respiratory tract (Figure 2(a)). Unlike the conjunctiva and nictitating membrane, viral antigen was barely detectable in the cornea. Interestingly, further IOD analysis showed that the nasolacrimal duct near the conjunctiva (region V) had more infected cells than the export in the vestibule (region I). It implied that the virus was drained by tear fluid in the nasolacrimal duct and attached to the epithelium (Figure 2(b)). Additionally, the positive cells were observed in different regions of the nasal cavity, with more staining cells in the respiratory region than in the olfactory region (Figure 2(b)). In regions II and III, where the nasal and nasolacrimal duct epithelium are closely connected, infected cells were also observed (*Supplementary Figure 1*). H1N1 infection in ocular and respiratory tissue was confirmed by western blot analysis (Figure 2(c)). Notably, the viral RNA and protein were detectable in blood cells within 6 hr after inoculation (Figures 2(c), *Supplementary Figure 1*). These results suggest that the nasolacrimal system serves as a route for virus migration to the respiratory tract.

### 3.3. Swine Influenza Virus Could Mount a Productive Infection following Ocular Inoculation in Pig

**Figure 3:**
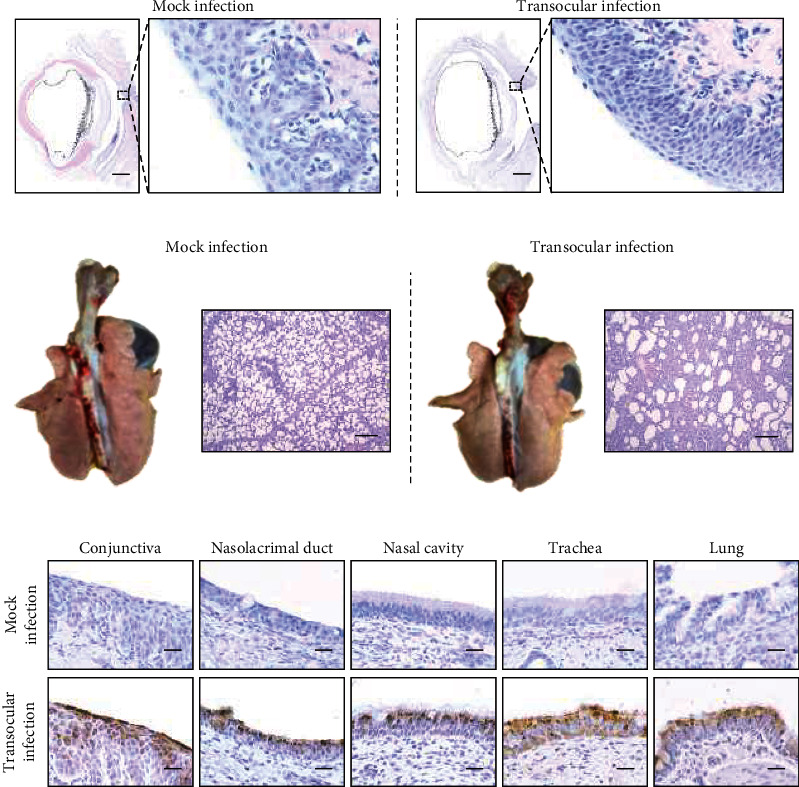
Tissue gross lesion, histopathological, and immunohistochemical examination. Conjunctiva and respiratory tract tissues were collected from piglets at 7 dpi. (a) No obvious pathological changes or organic damage were found in conjunctiva tissues from ocular inoculated piglets. Bar, 2,000 *μ*m (b) No obvious lung lesions were observed at necropsy. However, mild lung consolidation was observed in inoculated piglets. Bar, 200 *μ*m (c) H1N1-specific antigen could be observed in inoculated piglets. Bar, 20 *μ*m.

To confirm the productive infection via transocular infection, inoculated piglets were monitored daily for clinical signs and viral shedding. Inoculated piglets developed mild lethargy since 2 dpi, and sporadic sneeze was observed throughout the animal experiment period compared with mock inoculation. No fever was recorded in any animals. Nasal swabs were collected daily from the second and third groups to determine the virus shedding. At 7 dpi, we still can isolate the virus from nasal swab samples from 3/5 piglets (Table 2). No obvious lesion could be observed in conjunctiva or lung after necropsy, while congestion in vessels of alveolar walls could be observed in inoculated piglets. IHC examination identified H1N1-specific antigens in the conjunctiva, nasal mucosa, nasolacrimal duct, trachea, and lung of inoculated animals (Figures 3(a), 3(b), and 3(c). In the conjunctiva, more infected cells were observed in the epithelium of the eyelid margin and nictitating membrane. This is probably because there are fewer numbers of goblet cells in these areas which might resist the IAV by secreting mucus [15] (*Supplementary Figure 2*). Besides, the virus was isolated from the tracheas and lungs of all inoculated animals (Table 2). Although viral RNA and protein could be detected in conjunctiva in inoculated animals, we can only isolate the virus from the conjunctiva of 3/5 animals, which might be due to the low virus load. Overall, these results indicated that the H1N1 could establish a productive infection after ocular inoculation.

### 3.4. Both *α*-2,3-Linked and *α*-2,6-Linked SAs were Abundant in the Ocular Surface

**Figure 4:**
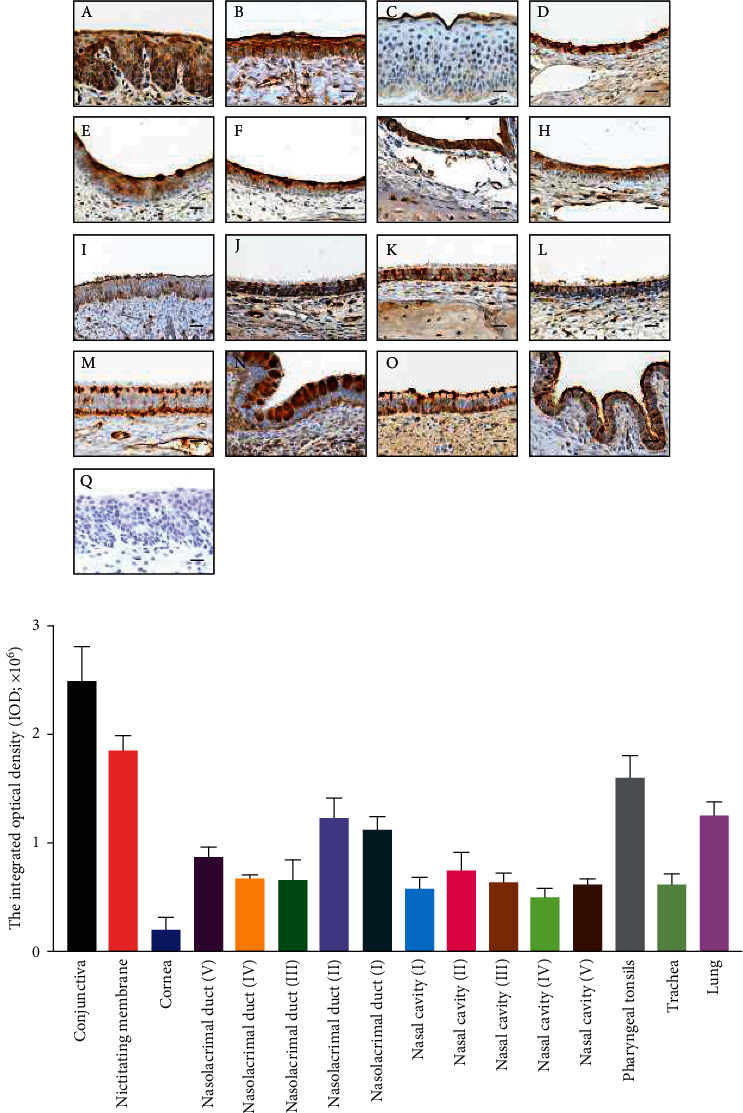
Distribution of *α* 2, 6-linked SA in different tissues. (a) SNA lectin labeling the *α* 2, 6-linked SA in different tissues. (A) conjunctiva; (B) nictitating membrane; (C) cornea; (D–H) nasolacrimal duct (V–I); (I–M) nasal cavity (I–V); (N) pharyngeal tonsils; (O) trachea; (P) lung; (Q) conjunctiva tissue pretreated with 5 mU of neuraminidase was used as a negative control. Bar, 20 *μ*m. (b) The average integrated optical density (IOD) of *α* 2, 6-linked sialic acid was obtained by analyzing three panoramic scans of different depth slides using ImageJ. All data shown are the mean results ± SD from three independent experiments.

**Figure 5:**
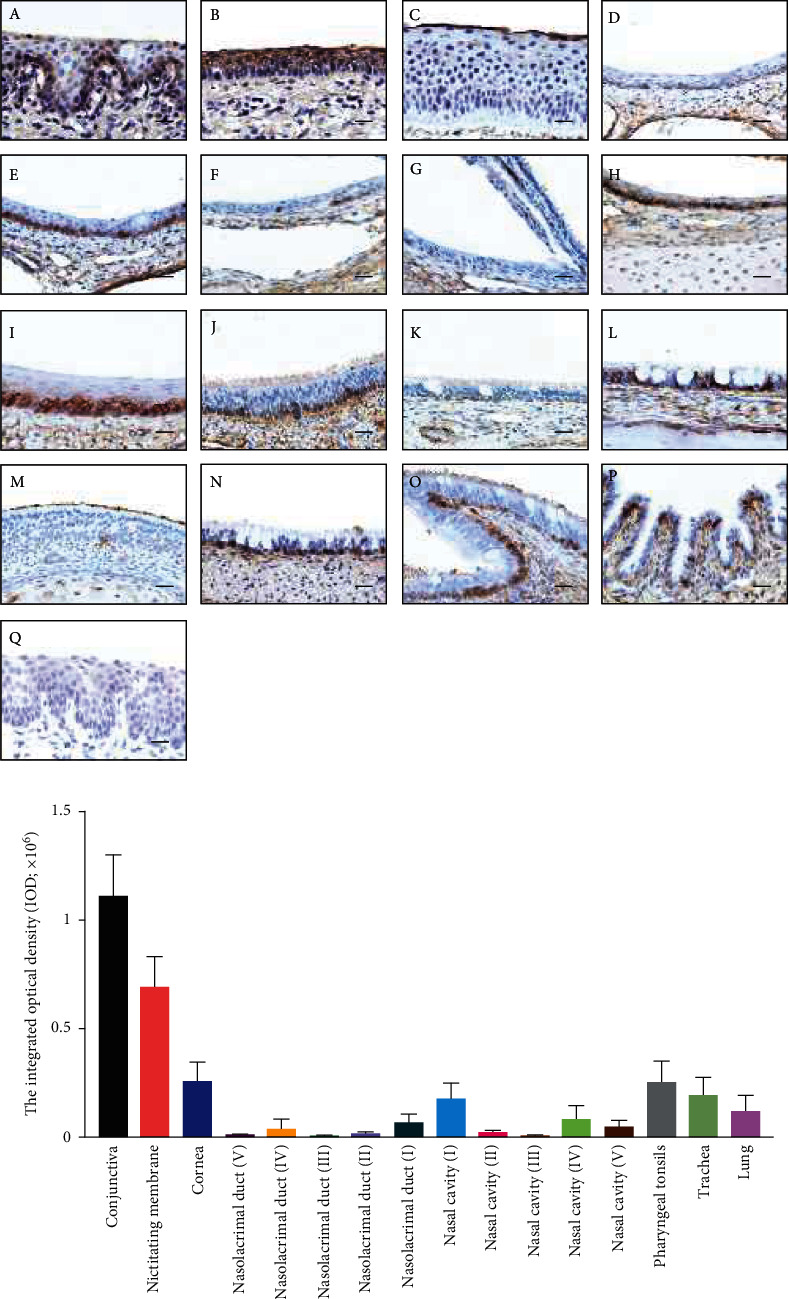
Distribution of *α* 2, 3-linked SA in different tissues. (a) MAII lectin labeling the *α* 2, 3-linked SA in different tissues. (A) conjunctiva; (B) nictitating membrane; (C) cornea; (D–H) nasolacrimal duct (V–I); (I–M) nasal cavity (I–V); (N) pharyngeal tonsils; (O) trachea; (P) lung; (Q) conjunctiva tissue pretreated with 5 mU of neuraminidase was used as a negative control. Bar, 20 *μ*m. (b) The average integrated optical density (IOD) of *α* 2, 3-linked sialic acid was obtained by analyzing three panoramic scans of different depth slides using ImageJ. All data shown are the mean results ± SD from three independent experiments.

SAs in the cell surface serve as the primary receptor for the influenza virus, while the *α*-2,3-linked and *α*-2,6-linked SA are preferentially bounded by avian and mammal influenza viruses, respectively [32]. To investigate the SA distribution in ocular tissues, lectins from MAL II and SNA were used to label the *α*-2,3-linked and *α*-2,6-linked SA, respectively. The conjunctiva and nictitating membrane could be stained by both lectins with no obvious staining in the cornea (Figures 4(a), 4(b), and *Supplementary Figure 3*). Moreover, the *α*-2,6-linked SA was widely distributed in different regions (I to V) of the nasolacrimal duct and the nasal passage epithelium (Figure 4(a) and *Supplementary Figure 3*). The *α*-2,3-linked SA seemed to distribute in the nasal cavity and nasolacrimal duct unevenly, and the specific staining could only be observed in regions I and IV (Figures 5(a) and 5(b). Both SAs were distributed in pharyngeal tonsils, trachea, and lung, which corresponded with previous studies. These results indicated that the porcine ocular surface has a great abundance of both SAs, which probably allows IAVs to invade.

### 3.5. Both Swine and Avian IAV Replicate in *Ex Vivo* Cultures of Porcine Conjunctiva

**Figure 6:**
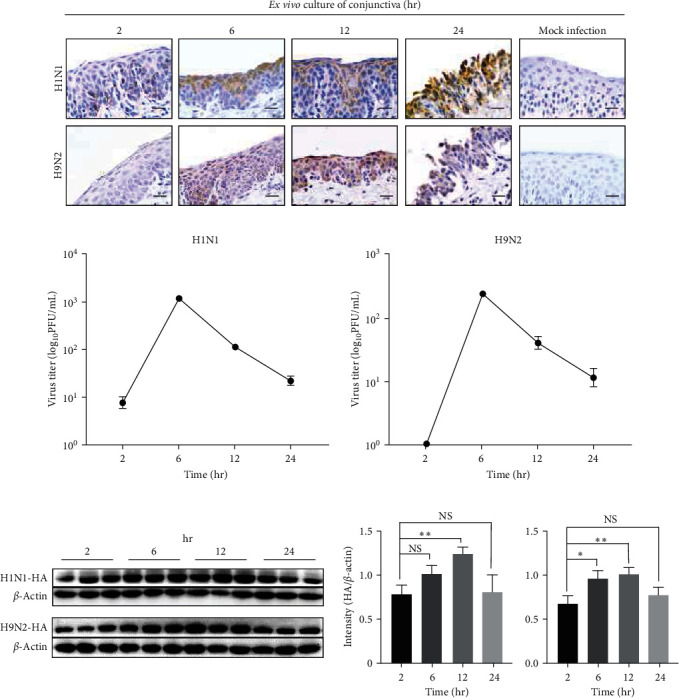
*Ex vivo* porcine conjunctiva culture supports the productive IAV infection. *Ex vivo* cultures of conjunctiva were incubated with H1N1 or H9N2 at 5 × 10^3^ TCID_50_. The tissues were collected for IHC staining or western blot analysis and the supernatants were used to perform plaque assay at different time points. (a) Viral antigens were detected in the ex vivo culture using IHC. Positive cells were stained brown. Bars: 20 *μ*m. (b) Viral titer in culture supernatant of *ex vivo* culture was determined by plaque assay on MDCK. Each error bar represents the mean and SD of three independent experiments. (c) Protein expression of H1N1 HA-protein in *ex vivo* culture at different time points detected by western blotting. The intensity (HA/*β*-actin) of H1N1-HA (left) and H9N2-HA (right) was measured using ImageJ. All data shown are the mean results ± SD from three independent experiments. Statistical significance was using one-way ANOVA. NS, no significance.  ^*∗*^*P* < 0.05,  ^*∗∗*^*P* < 0.01.

We found that porcine conjunctiva co-expressed the *α*-2,3-linked and *α*-2,6-linked SAs in this study. However, it needs to be confirmed whether the porcine conjunctiva could support productive viral replication of mammalian and avian IAV. Thus, the porcine conjunctiva explants were prepared and incubated with H1N1 and H9N2, respectively. For the H1N1 infection culture, viral antigens were detected at different time points with obvious epithelial damage at 24 hpi (Figure 6(a)). Western blot analysis showed that the viral protein increased at 6 and 12 hpi, but decreased at 24 hpi (Figure 6(c)). Whereas, progeny virions in the supernatant of *ex vivo* culture were dramatically increasing at 6 hpi but decreasing at 12 and 24 hpi (Figure 6(b)). Resemble productive infection was observed in the H9N2 infected culture (Figures 6(a), 6(b), and 6(c)). These results demonstrated that the IAV could attach and replicate in the porcine conjunctiva.

## 4. Discussion

Generally, IAVs are primarily transmitted via the respiratory route and initially infect the nasopharynx causing respiratory symptoms [33]. Certain subtypes of IAVs, such as H7, exhibit ocular tropism in humans, with sporadic studies reports of conjunctivitis caused by the H5 subtype [8, 9, 11, 19]. Additionally, H1N1 has been shown to infect human conjunctiva resulting in acute conjunctivitis and corneal erosions [7]. Previous studies reveal that a high titer infection could also be established after ocular exposure to IAVs [8, 16, 21]. These studies suggested that the ocular route cannot be ignored during the influenza pandemic. Figuring out how IAVs exploit the ocular surface to invade the host is crucial for assessing the potential risks of transocular infection [19]. Here, we proved that H1N1 could rapidly transmit to the respiratory tract via the nasolacrimal duct following ocular exposure.

Given the important role of SA in IAV attachment, it is warranted to investigate the SA distribution on the ocular surface [34]. Previous studies indicated that porcine is a “mixing vessel” for IAV due to the presence of both SAs in the respiratory tract [25]. Here, lectin labeling showed that both *α*-2,6-linked and *α*-2,3-linked SA are also widely expressed on the conjunctiva and nictitating membrane but not in the cornea of pigs. Moreover, the *ex vivo* culture of the conjunctiva supported the productive infection of H1N1 and H9N2, suggesting that IAV can attach to the SA in the conjunctiva to enter the host cells. Nevertheless, future studies should incorporate a broader range of IAV subtypes to verify the sensitivity of the porcine conjunctiva to different IAVs. Moreover, the *in vivo* study demonstrated that H1N1 was primarily observed in the conjunctiva and nictitating with no detection of the cornea which was sensitive to IAV in humans [15]. A substantial presence of viral antigens was observed in the eyelid margin where the goblet cells are absent. However, it remains unclear whether the mucus secreted by goblet cells in tear film plays a role in resisting the IAV.

In contrast to the respiratory tissue, the nasolacrimal duct is often overlooked in influenza research. Some studies speculated that the nasolacrimal system serves as a fast route to transport the virion to the respiratory tract from the conjunctiva [16, 17, 19]. Here, the structural characteristic of the porcine nasolacrimal duct was revealed by serial slices from the eye to the nasal vestibule. Viral nucleic acid could be detected in nasal swabs as early as 5 min in pigs after ocular inoculation, which corresponds with the study in human ocular exposure to aerosols containing the influenza virus [35]. Previous studies primarily focused on the infection and transmission following IAV ocular exposure, while the infection of the nasolacrimal duct had not been reported [16, 36]. In nasolacrimal duct obstruction cases, many common respiratory viruses, including coronavirus 229E, HKU1, OC43, and RSV, were detected in lacrimal tissue collected by surgery [37]. In this study, we detected the viral antigens in the initial (6 hpi) and acute phases (7 dpi) of infection, which demonstrated that the H1N1 could harbor in the epithelium of the nasolacrimal duct. These results first provide evidence that H1N1 could utilize the nasolacrimal systems to transfer into the respiratory tract after ocular exposure. Although viremia induced by pandemic influenza A/H1N1/2009 is an indicator of disease severity in humans, few studies report the characteristics of viremia caused by H1N1 in swine [38, 39, 40]. In this study, the H1N1 was detected in blood cells as early as 30 min following ocular inoculation, which is probably attributed to the nasolacrimal duct surrounded by cavernous vascular system [41]. However, further comparison with different inoculation routes, such as intranasal or intratracheal inoculation, is warranted.

Due to the high abundance of *α*-2,3-linked and *α*-2,6-linked SA on the ocular surface, it appears that IAV might utilize the ocular surface as an alternative route to spread in the swine herd. Though H1N1 infection in pigs typically results in low lethality rates, the spread of H1N1 poses a significant risk to public health safety [42, 43, 44]. In China, pig farmers have been found to have a high positive rate of H1N1 influenza serological antibodies [45, 46]. Human ocular tissue has a great abundance of *α*-2,3-linked SA, which may explain the higher incidence of conjunctivitis associated with avian IAV infection [6]. However, human *ex vivo* ocular tissue or cell monolayers were sensitive to different subtypes of IAVs in previous studies [15]. Beyond the appropriate receptors, however, the functional balance of HA and neuraminidase (NA) protein has been reported as crucial for conjunctiva tropism of H1N1 [12]. Restrictive host factors in different tissues also hamper viral tropism, such as the ocular secretory mucins [32, 47]. For respiratory viruses, the impact of ocular exposure can easily be ignored because researchers are inclined to focus on direct complications. The ocular surface and nasolacrimal duct can be utilized by the virus for entry even when the virus itself does not cause ocular complications [7]. Thus, ocular protection is necessary in clinical or some occupational settings.

## 5. Conclusion

Together, this study provided solid evidence that the ocular surface and nasolacrimal duct can serve as a concealed entry site for respiratory viruses. We reinforce the necessity of eye protection in addition to respiratory protection during the pandemic.

## Figures and Tables

**Table 1:** Detection of viral RNA in nasal swabs at different time points via qRT-PCR.

Timing (min)	Transocular inoculation	Mock infection
0	0/5^*∗*^ (-)	0/5 (-)
5	5/5 (23.16)^#^	0/5 (-)
10	4/5 (25.50)	0/5 (-)
30	3/5 (26.93)	0/5 (-)
60	2/5 (28.77)	0/5 (-)
120	4/5 (25.88)	0/5 (-)
360	3/5 (28.15)	0/5 (-)

^*∗*^Nasal swabs were analyzed for viral RNA by qRT-PCR. Threshold cycle (Ct) values lower than 30 were considered positive. Samples in which fluorescence was undetectable were considered negative. “x/5” indicates that the virus was detected in x of five pigs in the group. ^#^The number in brackets indicates the average mean Ct values of nasal swab samples, which are considered positive. “ - ” indicates that no viral RNA were detected in the samples.

**Table 2:** Virus replication in nasal swabs and organs of vaccinated or control pigs.

Group	Virus replication in nasal swabs or organs									
	Days postinoculation							Conjunctiva	Trachea	Lung
	1	2	3	4	5	6	7			
Transocular inoculation	5/5^*∗*^	5/5	5/5	2/5	3/5	3/5	3/5	3/5^#^	5/5	5/5
Mock inoculation	0/5	0/5	0/5	0/5	0/5	0/5	0/5	0/5	0/5	0/5

^*∗*^The virus in nasal swabs or organs were determined in eggs. The allantoic fluids was harvested after incubation at 37°C for 72 hr and then tested for hemagglutinin activity. “x/5” indicates that the virus was detected in x of five pigs in the group.

## Data Availability

The data used to support the findings of this study are included in the article.

## Supplementary Materials

Supplementary 1 Figure 1: IHC detected the viral antigen in the submucosal of the nasolacrimal duct and nasal passage.

Supplementary 2 Figure 2: panoramic screening of conjunctiva section performed via IHC targeting H1N1-HA.

Supplementary 3 Figure 3: SNA labeling of ocular tissues and different regions of nasal passage and nasolacrimal duct epithelium.
