# Supplementary material for: Influenza A Virus Utilizes the Nasolacrimal System to Establish Respiratory Infection after Ocular Exposure in the Swine Model
Source: Transbound Emerg Dis. 2024 Jun 27;2024:8192499. doi: 10.1155/2024/8192499 (PMC12016754; doi:10.1155/2024/8192499)
Supplement: Supplementary 1 — Figure 1: IHC detected the viral antigen in the submucosal of the nasolacrimal duct and nasal passage. [file 8192499.f1.docx]

**Mock infection**

**Transocular infection**


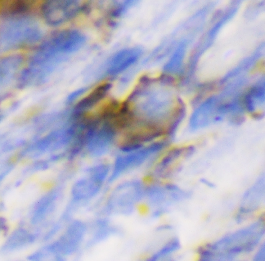

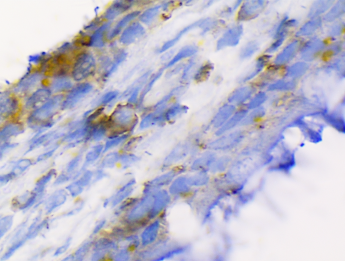


**Nasal cavity**

**Nasolacrimal duct**


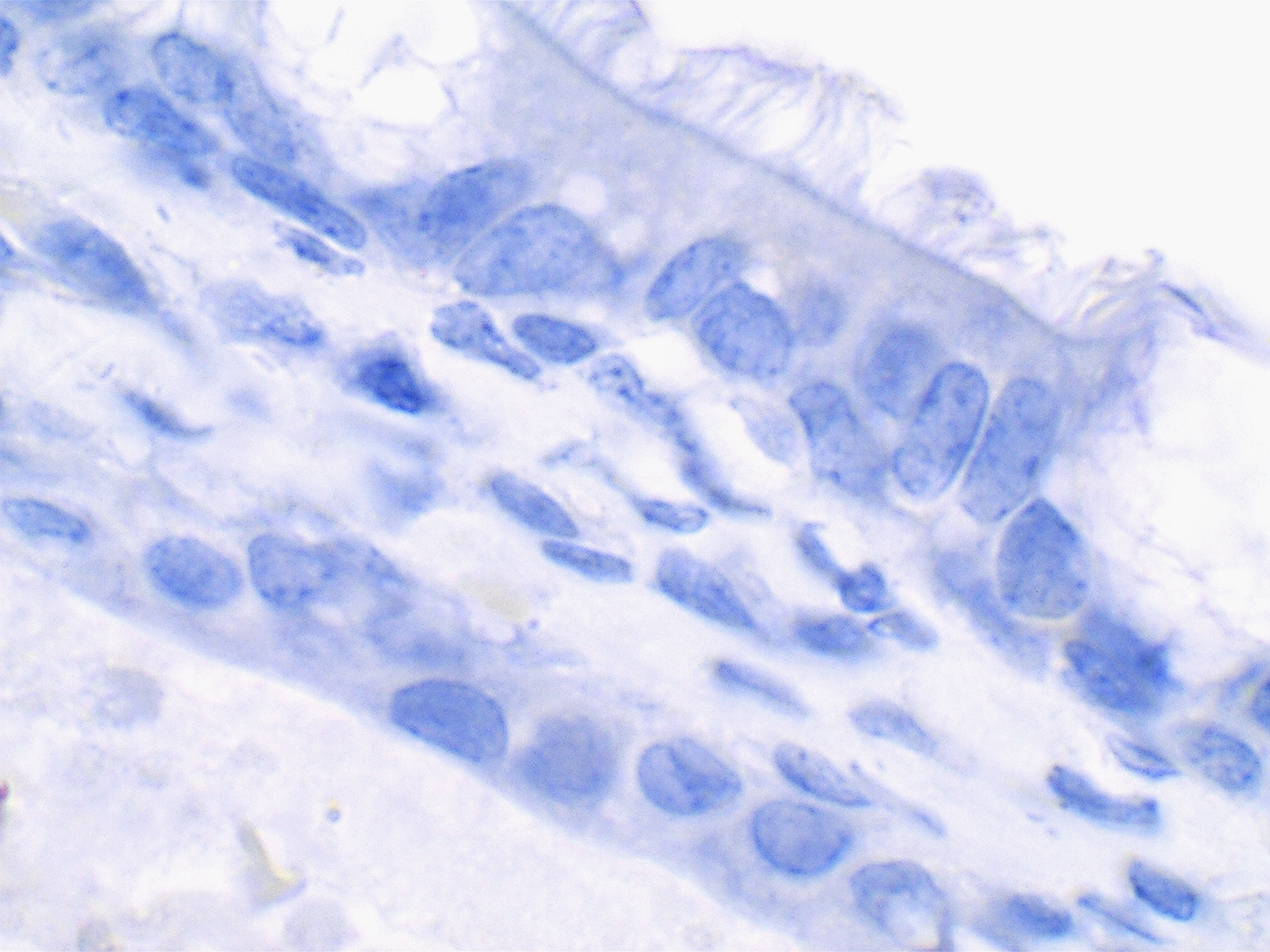

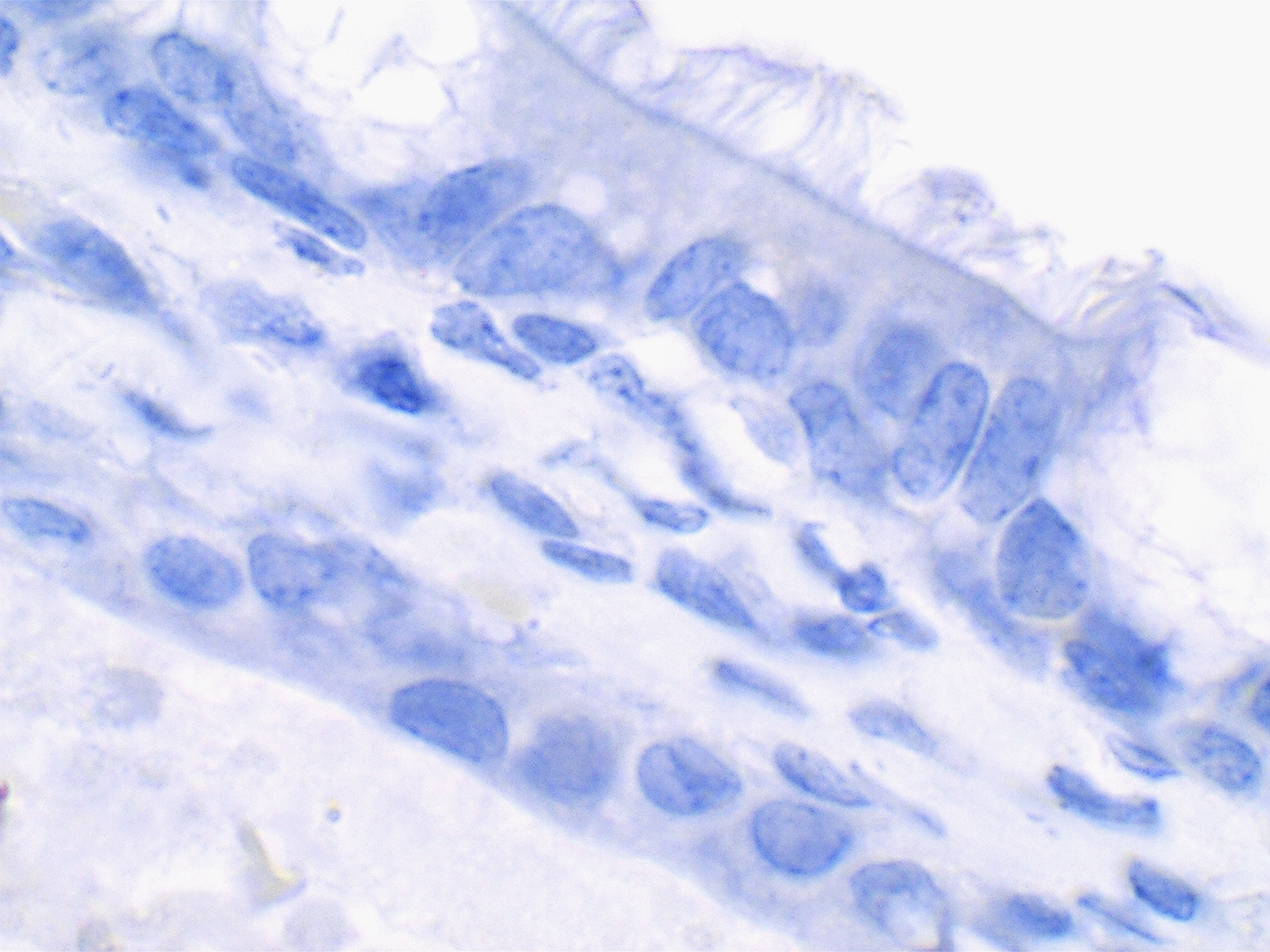


**Nasolacrimal duct**

**Nasal cavity**

Figure. S1

Figure S1 H1N1 enters the bloodstream after ocular inoculation. (A) At 6 hpi, H1N1-positive cells were detected in the submucosa between the nasal cavity and the nasolacrimal duct. Bar, 10 μm; (B) Detection of viral HA-gene in hemocytes from ocular inoculated piglets at different time points by reverse transcription-polymerase chain reaction (RT-PCR); (C) Detection of viral HA-protein in hemocytes from ocular inoculated piglets at different time points by Western blot.

A

C


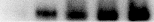

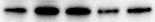


β-actin

HA

**0 h**

**0.5 h**

**2 h**

**4 h**

**6 h**

B


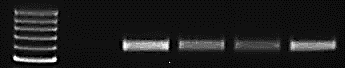


600bp

**Marker**

**0 h**

**0.5 h**

**2 h**

**4 h**

**6 h**
