# Supplementary material for: Influenza A Virus Utilizes the Nasolacrimal System to Establish Respiratory Infection after Ocular Exposure in the Swine Model
Source: Transbound Emerg Dis. 2024 Jun 27;2024:8192499. doi: 10.1155/2024/8192499 (PMC12016754; doi:10.1155/2024/8192499)
Supplement: Supplementary 2 — Figure 2: panoramic screening of conjunctiva section performed via IHC targeting H1N1-HA. [file 8192499.f2.docx]

Figure. S2

Figure S2 Transocular inoculated piglets were sacrificed at 7dpi and sagittal eye sections were prepared for IHC examination targeting H1N1 antigen. (a) tarsal conjunctiva; (b) Nictitating membrane; (c) Eyelid margin; (d) Cornea. The bar of the picture is 2000 μm of the middle picture and 20 μm of the others.


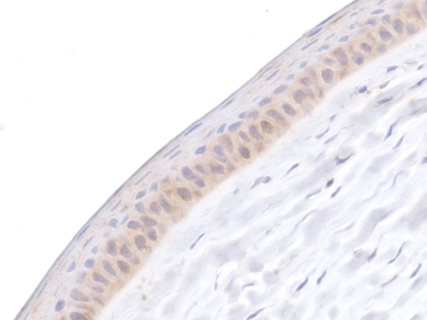

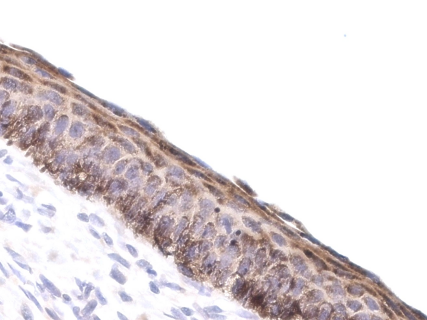

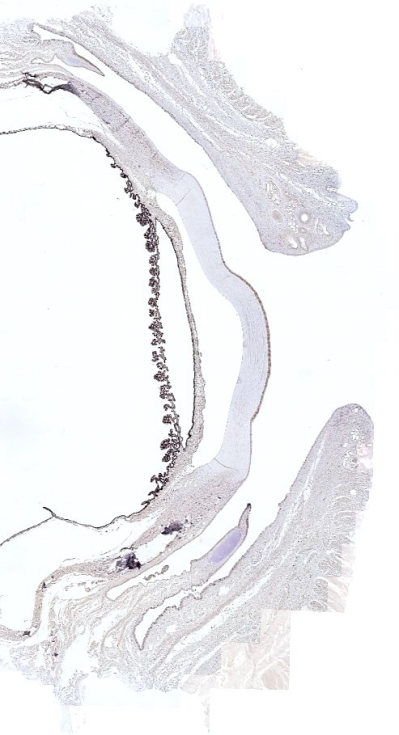

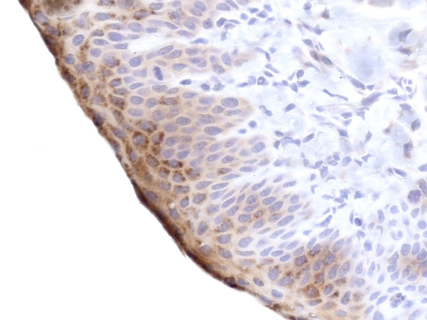

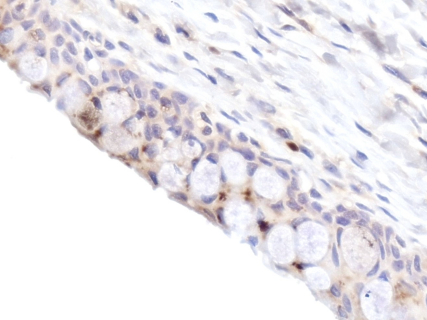


**d**

**b**

**a**

**c**
