# Supplementary material for: Influenza A Virus Utilizes the Nasolacrimal System to Establish Respiratory Infection after Ocular Exposure in the Swine Model
Source: Transbound Emerg Dis. 2024 Jun 27;2024:8192499. doi: 10.1155/2024/8192499 (PMC12016754; doi:10.1155/2024/8192499)
Supplement: Supplementary 3 — Figure 3: SNA labeling of ocular tissues and different regions of nasal passage and nasolacrimal duct epithelium. [file 8192499.f3.docx]

Figure. S3


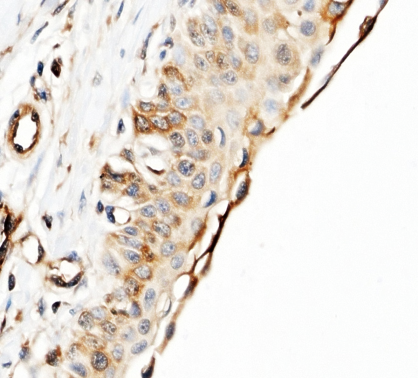

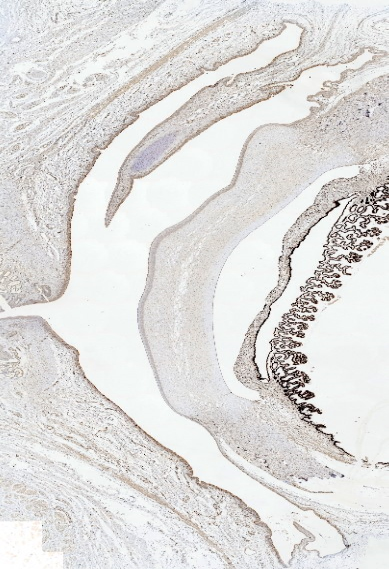


v

v

v

v

**a**

**b**

**d**

**c**


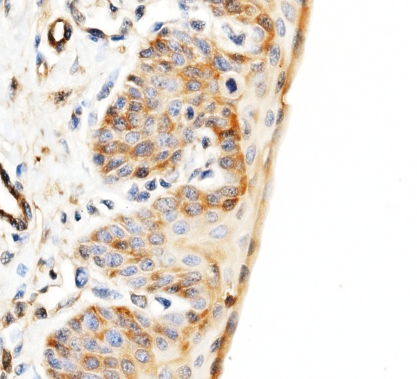

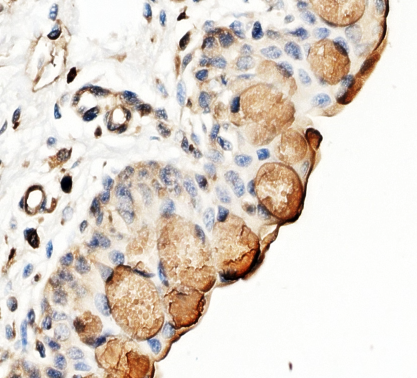

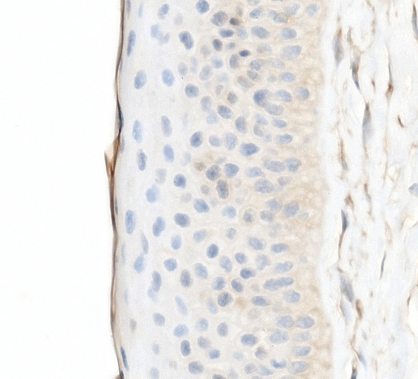


A

**a**

**c**

**d**

**I**

**II**

**III**

**IV**

**V**


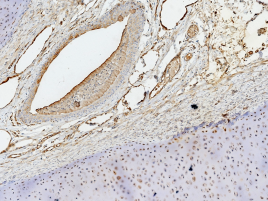

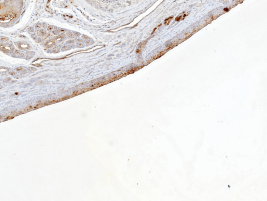

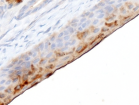

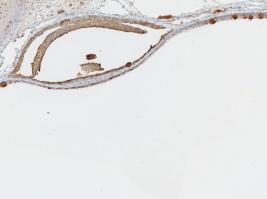

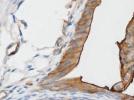

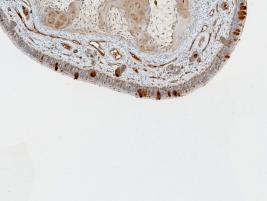

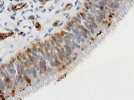

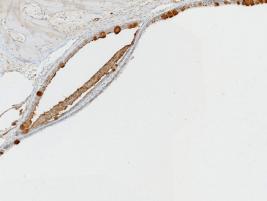

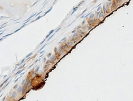

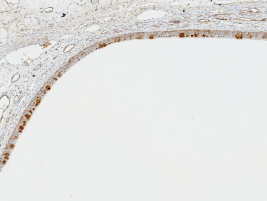

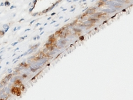

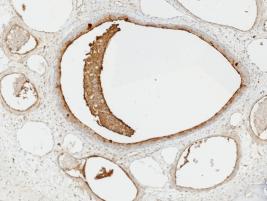

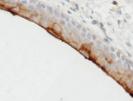

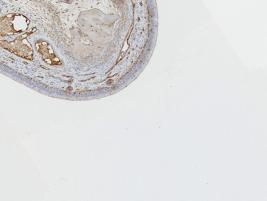

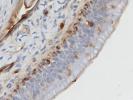

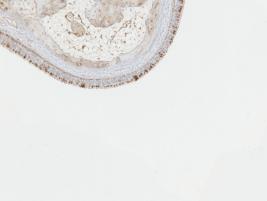

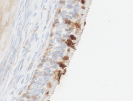

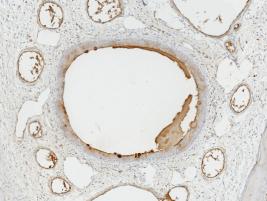

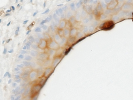

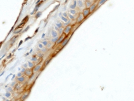


B

**Nasal cavity**

**Nasolacrimal duct**

Figure S3 (A) Panoramic scanning of porcine eye section stained by SNA: (a) tarsal conjunctiva; (b) Nictitating membrane; (c) Eyelid margin; (d) Cornea. The bar of the picture is 500 μm of the middle picture and 20 μm of the others. (B) IHC results showed the distribution pattern of α 2, 6-sialic acid in five cross-sections (I, II, III, IV, and V) of the piglet nasal cavity. The pictures at the upper were nasolacrimal duct, while the pictures at the bottom were nasal cavity.
